# Editorial: Strategies for preventing early childhood caries

**DOI:** 10.3389/froh.2026.1785571

**Published:** 2026-01-28

**Authors:** Moréniké Oluwátóyìn Foláyan, Tshepiso Mfolo, Maha El Tantawi

**Affiliations:** 1 Early Childhood Caries Advocacy Group, University of Manitoba, Winnipeg, MB, Canada; 2 AFRONE Network, Alexandria University, Alexandria, Egypt; 3 Department of Child Dental Health, Obafemi Awolowo University, Ile-Ife, Nigeria; 4 Department of Community Dentistry, University of Pretoria, Pretoria, South Africa; 5 South African Association of Paediatric Dentistry, Pretoria, South Africa; 6 Faculty of Dentistry, Alexandria University, Alexandria, Egypt; 7 Faculty of Dental Medicine, Alamein International University, New Alamein City, Egypt; 8 Egyptian Oral Health Association, Alexandria, Egypt

**Keywords:** health policy, health promotion, integrated health care, maternal and child care, patient-centered care (MeSH term), prenatal care (MeSH), primary prevention [MeSH], social determinants of health

For decades, Early Childhood Caries (ECC) has been recognized as one of the most common yet preventable chronic diseases of childhood (1), with implications for pain, growth, development, school readiness, and quality of life (2). Evidence shows that ECC is multifactorial, entrenched in a complex web of biological factors (e.g., *Streptococcus mutans* transmission, dietary sugars), behavioural practices (feeding habits, oral hygiene), and overarching social determinants of health, including socioeconomic status, parental education, and access to care (3) with debilitating effects when left untreated (4). Despite this, global public health and clinical responses have remained disproportionately curative, focusing on restorative treatment after the disease occurs, often under general anaesthesia for severe cases (5, 6). While interventions like silver diamine fluoride (SDF) offer a minimally invasive, cost-effective care (7), and prenatal oral health education is celebrated for offering prevention in a critical window (8), their integration into systemic, cross-disciplinary health strategies has been fragmented and inadequate. A persistent gap exists in translating our understanding of disease aetiology into scalable, equitable, and family-centered preventive frameworks in the first 1,000 days of life from pregnancy to age 2.

This Research Topic of five articles represents a significant stride toward filling this gap, collectively arguing for a foundational reorientation of ECC management. First, two papers leverage digital and communication technologies to expand reach and refine messaging. Lee et al. demonstrate that social media like Instagram have potential as powerful, low-cost platforms to disseminate evidence-based oral health promotion directly to caregivers, addressing the information-access gap. Complementing this, Crystal et al. present a qualitative analysis of parental feedback on SDF therapy that provides crucial insights into the patient-provider encounter. Their work moves beyond clinical efficacy to address communication barriers, parental concerns, and shared decision-making, ensuring that innovative tools are accepted and utilized effectively.

Second, two contributions deepen our understanding of implementation within real-world community and social contexts. Patano et al. analysed the feedback from multiple stakeholders (caregivers, community health workers, dentists) on a behavioural intervention for caregivers of young children undergoing surgery to treat severe ECC. Their analysis underscores the necessity of culturally tailored, feasible, and multi-level interventions. It highlights the indispensable role of community health workers in bridging clinical and home environments. The epidemiological study by Foláyan et al. reinforces the link between parental educational status and ECC, even within a specific local context, reminding us that upstream socioeconomic factors must be explicitly targeted in any comprehensive strategy to prevent ECC.

Finally, the commentary by Foláyan et al. on the first 1,000 days of life and the ECC global data gap serves as a crucial synthesizing and agenda-setting piece. It compellingly argues for positioning oral health as an integral component of early childhood development and maternal-child health programs. By identifying the scarcity of reliable and updated data during this primordial period, the commentary calls for a systemic overhaul of surveillance and research to match the biological and social complexity of ECC.

Together, these papers add dimensions to the field: they validate novel delivery channels (social media, teledentistry), emphasize human-centered design in clinical and behavioural interventions, reaffirm the centrality of social determinants, and issue a clarion call for integrated, data-driven action starting in pregnancy. The evidence is no longer simply calling for a shift toward prevention but mapping the pathway for shifting the paradigm from surgical repair to holistic prevention of ECC.

The next steps are clear. Oral health must be a mandated component of prenatal care packages, well-child visits, and public health nursing programs. Reimbursement models must incentivize preventive counselling and interventions like SDF application. Leveraging non-dental professionals (paediatricians, midwives, community health workers, dietitians) for task sharing (9) requires standardized, interdisciplinary training and clear referral pathways. National and global health tracking systems must integrate core oral health indicators into maternal and child health surveillance systems to close the data gap. Building on the Instagram study, public health agencies must collaborate with communication experts to launch coordinated, evidence-based, and anti-stigma campaigns that reframe ECC as a preventable non-communicable disease. Continuing to pour resources primarily into surgical repair of a preventable disease is ethically and economically unsustainable. These five papers light the way toward a future where every child's right to oral health is secured from the very first days of life, through informed families, supported by integrated healthcare systems, and guided by equitable policies. The paradigm must shift.
